# A Self-Localization Algorithm for Mobile Targets in Indoor Wireless Sensor Networks Using Wake-Up Media Access Control Protocol

**DOI:** 10.3390/s24030802

**Published:** 2024-01-25

**Authors:** Rihab Souissi, Salwa Sahnoun, Mohamed Khalil Baazaoui, Robert Fromm, Ahmed Fakhfakh, Faouzi Derbel

**Affiliations:** 1 Smart Diagnostic and Online Monitoring, Leipzig University of Applied Sciences, Wächterstraße 13, 04107 Leipzig, Germany; rihab.souissi@stud.htwk-leipzig.de (R.S.); robert.fromm@htwk-leipzig.de (R.F.); faouzi.derbel@htwk-leipzig.de (F.D.); 2 Laboratory of Signals, Systems, Artificial Intelligence and Networks (SM@RTS), Digital Research Center of Sfax (CRNS), Sfax University, Sfax 3021, Tunisia; salwa.sahnoun@enetcom.usf.tn (S.S.); ahmed.fakhfakh@enetcom.usf.tn (A.F.); 3 National School of Electronics and Telecommunications of Sfax, Sfax 3018, Tunisia

**Keywords:** indoor localization, wireless sensor network, received signal strength indication, low-energy consumption, wake-up receiver, OMNeT++

## Abstract

Indoor localization of a mobile target represents a prominent application within wireless sensor network (WSN), showcasing significant values and scientific interest. Interference, obstacles, and energy consumption are critical challenges for indoor applications and battery replacements. A proposed tracking system deals with several factors such as latency, energy consumption, and accuracy presenting an innovative solution for the mobile localization application. In this paper, a novel algorithm introduces a self-localization algorithm for mobile targets using the wake-up media access control (MAC) protocol. The developed tracking application is based on the trilateration technique with received signal strength indication (RSSI) measurements. Simulations are implemented in the objective modular network testbed in C++ (OMNeT++) discrete event simulator using the C++ programming language, and the RSSI values introduced are based on real indoor measurements. In addition, a determination approach for finding the optimal parameters of RSSI is assigned to implement for the simulation parameters. Simulation results show a significant reduction in power consumption and exceptional accuracy, with an average error of 1.91 m in 90% of cases. This method allows the optimization of overall energy consumption, which consumes only 2.69% during the localization of 100 different positions.

## 1. Introduction

Indoor localization systems are growing worldwide to track objects in challenging conditions. The development of applications depends on the challenges of the targets’ mobility, cost, environment, etc. Furthermore, the difficulties in ensuring the best precision of the designed system in internet of things (IoT) systems according to expected and unexpected conditions for predetermined areas have become a wide field of research [1].

Mobile positioning using global positioning systems (GPS) is widely used in outdoor areas with high accuracy, but it is limited for indoor environments due to the signal attenuation caused by obstacles in covered areas [2]. In this context, several indoor localization technologies have been introduced, such as frequency modulation (FM) [3], radio frequency identification (RFID) [4] and ultra wide band (UWB) [5]. The variety of these technologies is based on measurements such as RSSI [6], time of flight (TOF), Time Difference of Arrival (TDOA) [7] and angle of arrival (AOA) [8]. RSSI is the most popular, as it is easy to implement and the distance between the receiver and transmitter can be extracted from RSSI values using the path loss model of the RSSI formula. Estimation of the target position in indoor environments depends on the technique used, node deployment, state of communication between nodes, and node lifetime. Any communication interruption can reduce the accuracy of the application [9]. However, mobility is another challenge for localization applications. For these applications, sensors are the basis of architecture. Each sensor consists of a microcontroller, wireless transceiver, memory, and battery to power the node. The main challenge of indoor localization is the limited battery life of the sensors. High communication traffic during application depletes the battery [10]. Due to this high communication demand between nodes, the network lost nodes and coverage space for a short time. To reduce energy consumption for indoor localization, different studies for energy saving were investigated, such as routing protocols MAC protocols [11], genetic algorithm [12], wake-up receiver (WuRx) implementation [13].

The potential of this work is to combine such techniques to locate a mobile target. The contribution is to simulate a real network scenario for indoor localization applications with the implementation of software and hardware metrics in a simulator. A new simulation with real parameters is introduced. The main idea of the proposed application is to save energy consumption by reducing the maximum of communication and defining a matrix architecture with dynamic anchors to attain maximum accuracy. We proposed the implementation of a wake-up MAC protocol using a real model of energy consumption. We present a new indoor localization algorithm for a mobile target using the variation in transmission power to reach the closer anchors. To locate the target node, the proposed application used trilateration with the RSSI model. Therefore, an adaptation of RSSI metrics was required to extract the input values for the RSSI model. After a comprehensive implementation of all relevant metrics, a careful evaluation of the accuracy of the model is performed in a small area. Following the confirmation of our studies in a small area, we perform a simulation with the same inputs in a large area with specific architecture in terms of accuracy and energy consumption.

Section 2 provides an overview of the related background theory and state-of-the-art. Section 3 presents a definition of the hardware and the technique used for the localization application. In Section 4, we provide an explanation of our proposed method and the generality of the parameters used in our extended simulation. Section 5 presents the results of our simulations and analyzes the findings. Finally, the conclusions and future work are discussed in Section 6. The success of these applications depends on the development of a cost-effective and robust real-time system capable of accurately locating objects.

## 2. Related Works

Indoor Positioning Systems (IPSs) are real-time systems designed to accurately determine the location of agents or objects, either moving or stationary, within indoor environments. It is an advanced solution to track patients in medical environments [14], IoT systems [15], smart campuses [16], and other fields that need to track objects and monitor such areas. The accuracy of an indoor positioning system is significantly affected by a variety of factors and is highly dependent on the frequency range used and the robustness of the system to obstacles and interference in indoor environments. In this context, numerous studies have been conducted to evaluate the performance of different technologies faced with different challenges. Table 1 shows the performance of different technologies in different scenarios according to [17,18,19,20,21,22,23]. In [17], the authors conducted a comparative analysis between trilateration and min–max approaches. Their investigation focused on evaluating the effectiveness of Received Signal Strength Indication (RSSI) measurements based on ZigBee standards operating at a frequency of 2.4 GHz. This specific operating frequency is known to cover distances of approximately 1–3 m. The multipath interference generates unreliable RSSI values and causes an average error of 3.6 m, which is higher compared to the tested area. In [18], a practical implementation of a Bluetooth Low Energy (BLE)-based localization system was introduced. The used system integrates multilateration and Kalman filtering techniques to provide a low-cost solution while maintaining high positional accuracy. The authors achieved an average error of about 2.33 m for a tested area of 9.77 m × 13.45 m.

The authors of [19] present a comprehensive comparison of various IoT communication technologies, including Wi-Fi, Bluetooth Low Energy (BLE), ZigBee, and LoRaWAN. The results of their study showed significant improvements in positioning accuracy by 4% with BLE. BLE showed an improvement of 0.719 m, Wi-Fi improved by 0.517 m, LoRaWAN showed 0.793 m, and ZigBee improved accuracy by 0.741 m. Despite the effectiveness of Wi-Fi, which presents the best error, it consumes substantial power, presenting a challenge when considering battery-powered devices. On the other hand, BLE emerges as the second most accurate technology, with a low energy consumption. However, it suffers from a limited transmission range compared to other devices tested. LoRaWAN is impressive long-range capabilities make it a potentially cost-effective technology. It can be a required solution for large-scale coverage by minimizing the number of devices needed to create and maintain a reliable network. However, its relatively high power consumption can be a challenge, especially in energy-saving scenarios. In [20], authors show other results by comparing UWB using TOF, and Wi-Fi with RSSI. The impressive error rate was the result of UWB with TOF with 1.56 m. In [21], the authors used the UHF radio frequency identification RFID system to estimate the indoor position of a passive tag utilizing a received signal strength indicator (RSSI) with KNN to attend 0.18 m. For [22], the authors used infrared data from a low-resolution 16-pixel thermopile sensor array for a room 3 m × 3 m. The researchers conducted experimental studies to track individuals in an indoor environment. They performed comparative analyses between 1D-CNN and LSTM models with an (RMSE) of 0.096 m. The authors in [23] present a system that utilizes an ultrasound array to transmit chirp signals and employs time-of-flight measurement for ranging. The receiver position is estimated iteratively using the spring relaxation technique. Comparative investigations have been carried out with the visible light positioning system, revealing a median error of 0.0337 m.

Research has extensively investigated novel algorithms using trilateration, as shown in studies such as [24,25], or multilateration algorithms such as [26]. However, energy consumption is overlooked in these studies, which is critical for real implementation. Energy consumption has a direct impact on the lifetime of the network. It is essential to find a balance between accuracy and energy consumption to ensure the possibility of localization algorithms in real-world scenarios. When evaluating algorithm performance, researchers should consider factors such as algorithm complexity, sensor activation, sleep mode, optimization techniques, hardware efficiency, environment conditions, and battery capacity. Other studies have focused on evaluating the energy consumption of network systems such as [12,13]. In [27], the authors studied the energy consumption of existing localization systems. They analyze both the energy consumption and the accuracy and they achieve a consumption of 0.8 Wh for a battery 3.7 V during 24 h. However, they presented an accuracy of 4.51 m.

In this work, we focus on minimizing energy consumption by using a wake-up MAC protocol and a new algorithm for indoor localization. Our algorithm used a variation in transmission power to wake the nearer anchors and save energy. We proposed a new grid architecture to reduce the number of nodes used and the cost of the network. All simulation was implemented in the OMNeT++ discrete event simulator using the C++ programming language. Additionally, we used real parameters in the energy model and optimal parameters of RSSI.

## 3. System Setup

### 3.1. Wake-Up Receiver

**Figure 1:**
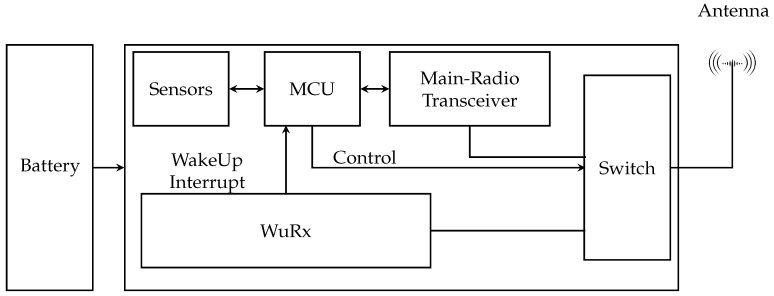
A wireless sensor node including WuRx hardware according to [28].

A WuRx is a specialized radio frequency (RF) receiver designed to maintain continuous reception mode for a sensor node. To achieve this, various approaches utilizing both passive and active components are employed to ensure that the WuRx’s power consumption remains below 10 μW. Figure 1 illustrates how a WuRx can be integrated into a sensor node.

As we can see, the node has two radio modules:

- Low-power radio receiver WuRx, which can only receive wake-up packet (WuPt) with an already specified pattern.
- Main radio transceiver, which has higher data transmission and reception capabilities.

Both radio modules share the same antenna. The microcontroller unit (MCU) gives one of these radio modules access to the antenna using the radio switch. When the node is powered on, it starts the WuRx listening mode: the MCU puts the main radio into sleep mode and switches the antenna to the activated WuRx.

**Figure 2:**
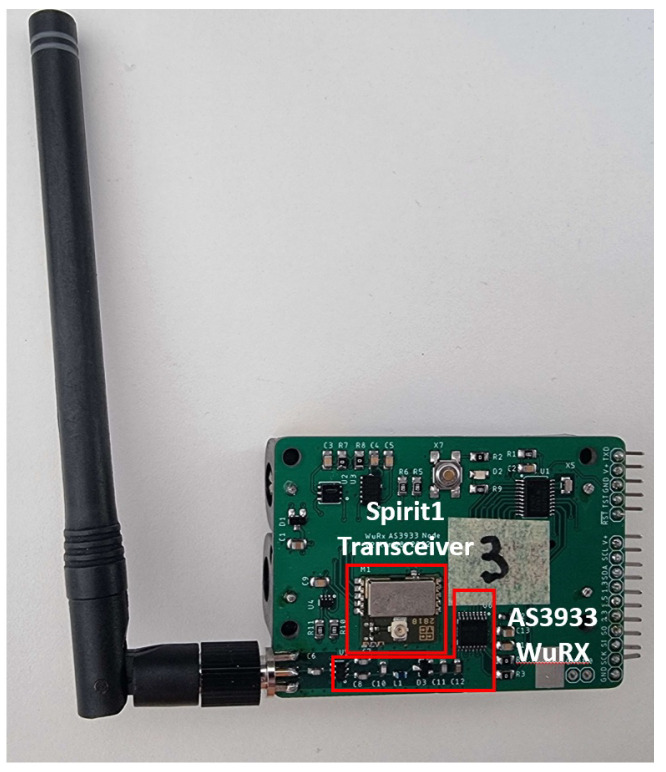
Wake-up receiver node.

The most powerful WuRx is the one that offers the best compromise between the amount of power consumed during idle listening mode and its sensitivity. The nodes used for the WuRx module are developed by the authors [29]. The various hardware specifications of the WuRx-based wireless node are elaborated in Table 2. In WuRx idle listening, the WuRx has a sensitivity of −61.6 dBm and consumes 5.71 μW. When the WuRx receives a WuPt that matches its address, it sends an interrupt to the MCU. At that moment, the MCU switches on the WuRx, turns on the various components that were in sleep mode or switched off, and switches the antenna to the main radio transceiver. Unlike the WuRx, the main-radio transceiver can perform both data transmission and reception, boasting a higher sensitivity level of up to −107 dBm. Figure 2 presents one of the nodes that we used in this work.

### 3.2. Received Signal Strength Indicator

RSSI is a well-known parameter in wireless communication networks that is used to measure signal strength and receive from wireless devices. RSSI value depends on the distance between the transmitter and the receiver. By contrast, the RSSI value decreases when increasing distance. Also, the RSSI value is susceptible to noise, multipath fading, and interference. These parameters can increase RSSI values. It is a value framed between 0 dBm and −100 dBm. An RSSI closer to 0 dBm presents the best signal and the short distance between the transmitter and the receiver. Referring to studies, RSSI uses logarithmic distance path loss with calculations based on equations shown in Equations (1) and (2) [30]

(1) $$RSSI_{d}=RSSI\left(d_{0}\right)-10*\eta *log\left(\frac{d}{d_{0}}\right)+X_{\sigma }$$

(2) $$d_{RSSI}=10^{\frac{\left(RSSI_{ref}-RSSI\right)}{10\eta }}$$

where:

- *d* is the distance between the transmitter and the receiver device.
- $d_{0}$ is the reference distance.
- $RSSI(d_{0})$ is the RSSI value obtained by the device at the distance $d_{0}$.
- $X_{\sigma }$ is the distributed noise Gaussian with an average of zero and a variance of $\sigma^{2}$.
- η is path loss exponents.

The value of η or the path loss exponent varies depending on the test environment. The value used for η in this equation ranges from 1.6 to 6 for the indoor environment and from 2.7 to 5 for the outdoor cases [31]. The detailed values are discussed in Table 3. RSSI closer to 0 dBm is the best and lower than −80 dBm is admitted but not useful for practical applications due to high noise [32]. Table 4 presents the signal strength acceptances according to the RSSI values.

### 3.3. Trilateration

**Figure 3:**
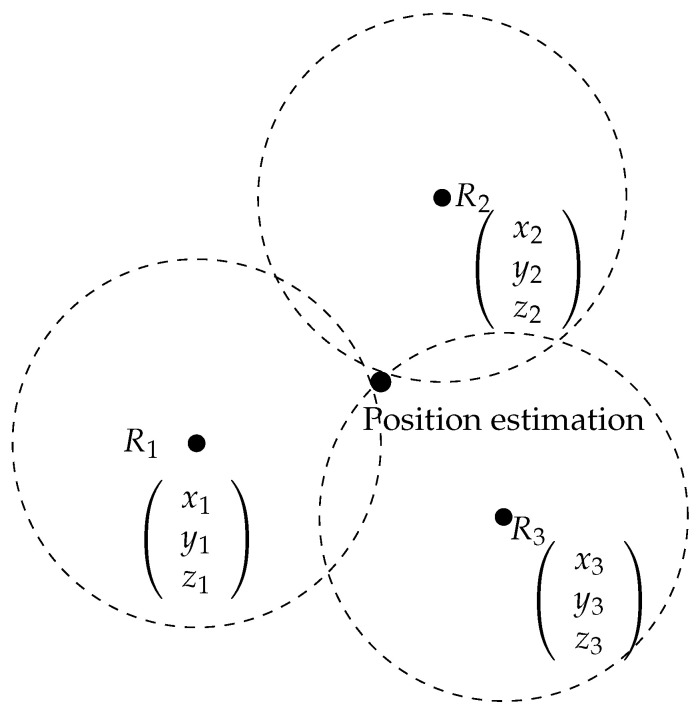
Schematic diagram of trilateration model.

Trilateration is a method for position determination through distance measurement using signal strengths. This algorithm is used in indoor positioning due to its accuracy and low cost, to find the position of an object based on three or more stations located in a known place. These positions are treated as the vertices of a triangle. The location of the object is obtained by solving a system of Equations (3) that contains the coordinates of the reference points $R_{1}$, $R_{2}$ and $R_{3}$ as presented in Figure 3 [33].

(3) $$\left\{\begin{array}{c} \left(x-x_{1}\right)+\left(y-y_{1}\right)+\left(z-z_{1}\right)=d_{1}\\ \left(x-x_{2}\right)+\left(y-y_{2}\right)+\left(z-z_{2}\right)=d_{2}\\ \left(x-x_{3}\right)+\left(y-y_{3}\right)+\left(z-z_{3}\right)=d_{3} \end{array}\right.$$

where $d_{i}$ is the distance between the target and each reference node. If we have 2D coordination, the distance will be as mentioned in Equations (4)–(6).

(4) $$d_{1}=\sqrt{{\left(x-x_{1}\right)}^{2}+{\left(y-y_{1}\right)}^{2}}$$

(5) $$d_{2}=\sqrt{{\left(x-x_{2}\right)}^{2}+{\left(y-y_{2}\right)}^{2}}$$

(6) $$d_{3}=\sqrt{{\left(x-x_{3}\right)}^{2}+{\left(y-y_{3}\right)}^{2}}$$

We can use Equations (4)–(6) to calculate the target position.

(7) $$d_{1}^{2}-d_{2}^{2}=-2x_{1}x-2y_{1}y-x_{2}^{2}-y_{2}^{2}+2x_{2}x+2y_{2}y+x_{1}^{2}+y_{1}^{2}$$

(8) $$d_{1}^{2}-d_{3}^{2}=-2x_{1}x-2y_{1}y-x_{3}^{2}-y_{3}^{2}+2x_{3}x+2y_{3}y+x_{1}^{2}+y_{1}^{2}$$

Equations (7) and (8) can be written in the system as following

(9) $$\left[\begin{array}{c} d_{1}^{2}-d_{2}^{2}+x_{2}^{2}+y_{2}^{2}-x_{1}^{2}-y_{1}^{2}\\ d_{1}^{2}-d_{3}^{2}+x_{3}^{2}+y_{3}^{2}-x_{1}^{2}-y_{1}^{2} \end{array}\right]=\left[\begin{array}{cc} 2(x_{2}-x_{1}) & 2(y_{2}-y_{1})\\ 2(x_{3}-x_{1}) & 2(y_{3}-y_{1}) \end{array}\right]\left[\begin{array}{c} x\\ y \end{array}\right]$$

When the number of reference nodes increases to N reference nodes we ameliorate the accuracy. We extend equations in system (9) to n equations:

(10) $$\left[\begin{array}{cc} d_{1}^{2}-d_{2}^{2}+x_{2}^{2}+y_{2}^{2}-x_{1}^{2}-y_{1}^{2}\\ d_{1}^{2}-d_{3}^{2}+x_{3}^{2}+y_{3}^{2}-x_{1}^{2}-y_{1}^{2}\\ \vdots & \vdots \\ d_{1}^{2}-d_{n}^{2}+x_{n}^{2}+y_{n}^{2}-x_{1}^{2}-y_{1}^{2} \end{array}\right]=\left[\begin{array}{ccc} 2(x_{2}-x_{1}) & 2(y_{2}-y_{1})\\ 2(x_{3}-x_{1}) & 2(y_{3}-y_{1})\\ \vdots & \vdots & \\ 2(x_{n}-x_{1}) & 2(y_{n}-y_{1}) \end{array}\right]\left[\begin{array}{c} x\\ y \end{array}\right]$$

Equation (10) can be written as the product of two matrices as follows: (11):

(11) $$B=A\left[\begin{array}{c} x\\ y \end{array}\right]$$

where

(12) $$B=\left[\begin{array}{cc} d_{1}^{2}-d_{2}^{2}+x_{2}^{2}+y_{2}^{2}-x_{1}^{2}-y_{1}^{2}\\ d_{1}^{2}-d_{3}^{2}+x_{3}^{2}+y_{3}^{2}-x_{1}^{2}-y_{1}^{2}\\ \vdots & \vdots \\ d_{1}^{2}-d_{n}^{2}+x_{n}^{2}+y_{n}^{2}-x_{1}^{2}-y_{1}^{2} \end{array}\right]$$

and *A* is

(13) $$A=\left[\begin{array}{cc} 2(x_{2}-x_{1}) & 2(y_{2}-y_{1})\\ 2(x_{3}-x_{1}) & 2(y_{3}-y_{1})\\ \vdots & \vdots \\ 2(x_{n}-x_{1}) & 2(y_{n}-y_{1}) \end{array}\right]$$

To calculate the solution of Equation (11), we derived Equation (14)

(14) $$\left[\begin{array}{c} x\\ y \end{array}\right]={\left(A^{T}A\right)}^{-1}A^{T}B$$

## 4. Proposed Algorithm for Mobile Target Localization

The accuracy of localization applications is a critical issue for indoor localization applications due to the high communication between nodes and interference with other signals. This problem is especially requested for large-scale WSN systems. In this regard, a new indoor localization application is applied using RSSI with a trilateration method. In this work, the energy consumption is optimized by using the wake-up MAC protocol.

### 4.1. Architecture of Proposed Grid

**Figure 4:**
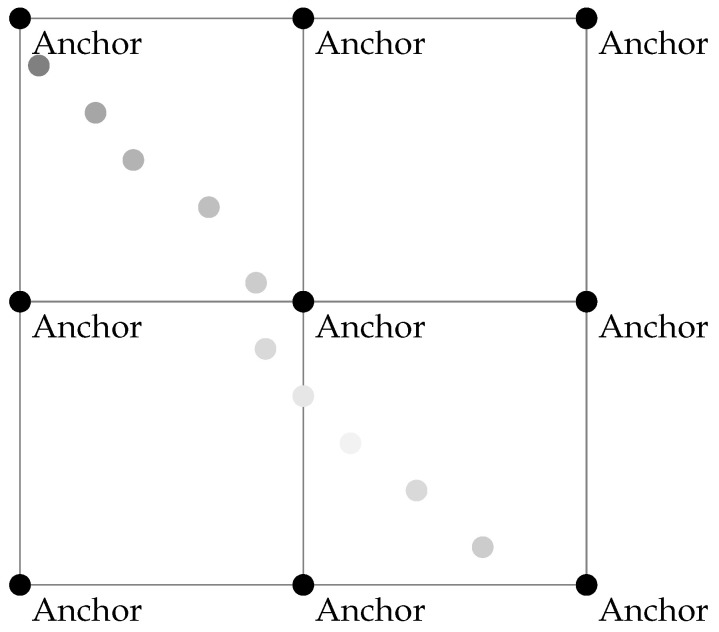
Proposed architecture.

The proposed algorithm is structured in grid form where a large rectangular area is neatly divided into multiple cells, as shown in Figure 4. Each corner of these cells is precisely marked by a point, and this point will be the position of the anchor node. These anchors serve as reference points, allowing systematic organization and positioning within the divided space. This design ensures that every part of the area is efficiently utilized, and the presence of anchors at each cell corner facilitates various applications such as spatial referencing or node placement in a grid-based system. The black degradation points are the different positions of the mobile target that can be taken inside the area. The goal of this architecture is to optimize the number of anchors used and to build a structure that covers all areas.

### 4.2. Wake-Up MAC Protocol

Tracking MAC protocols differs from other traditional MAC protocols. For tracking protocols, nodes turn to ON states if communication is established. The proposed MAC protocol was inspired by WuRx states.

It has two working states: the wake-up state and the sleep state. For the wake-up state, the radio transceiver is turned ON and the node can send or receive packets using minimum delay. If there is no activity of receiving or sending, the node keeps in a sleep state. After a sleep state, a node can turn ON in two cases:

- If it receives a demand from the upper layer. When it finishes transmitting the packet it turns OFF.
- If it receives a wake-up call from another node to wake up and start the application phase.

**Figure 5:**
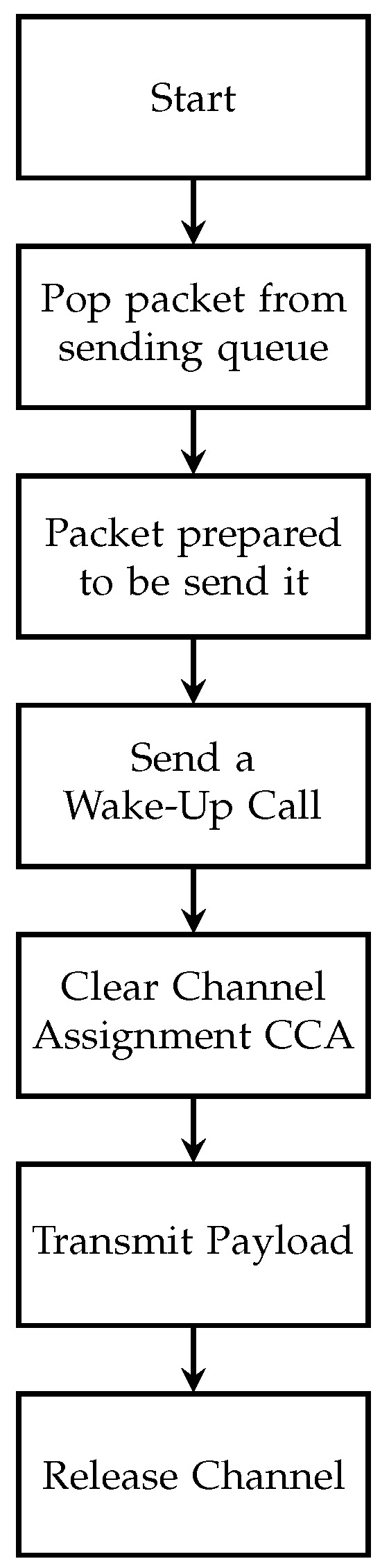
The flow chart for sending wake-up MAC protocol.

Wake-up MAC protocol uses a wake-up call to wake nodes. Then, a receiver node sends back an acknowledgment. A wake-up call contains the addresses of the receiver and sender. Attaching addresses to wake-up calls helps to prevent overheating problems. The flow chart of the wake-up MAC protocol is detailed in Figure 5.

### 4.3. Proposed Application

Taking advantage of dynamic anchors and adaptive transmission power control is a strategic approach aimed at maximizing both accuracy and resource efficiency. In this method, the anchors are in fixed positions, and the selection to wake the nearer anchors is adjusted in response to the target’s movements by variation in transmission power, thereby reducing the number of active anchors while maintaining precise localization capabilities. Meanwhile, varying transmitted power ensures that communication is optimized for the target’s proximity, balancing energy conservation with increased accuracy. By seamlessly adapting to changing conditions and target locations, this approach provides a compelling solution for achieving reliable and efficient localization in dynamic environments.

**Figure 6:**
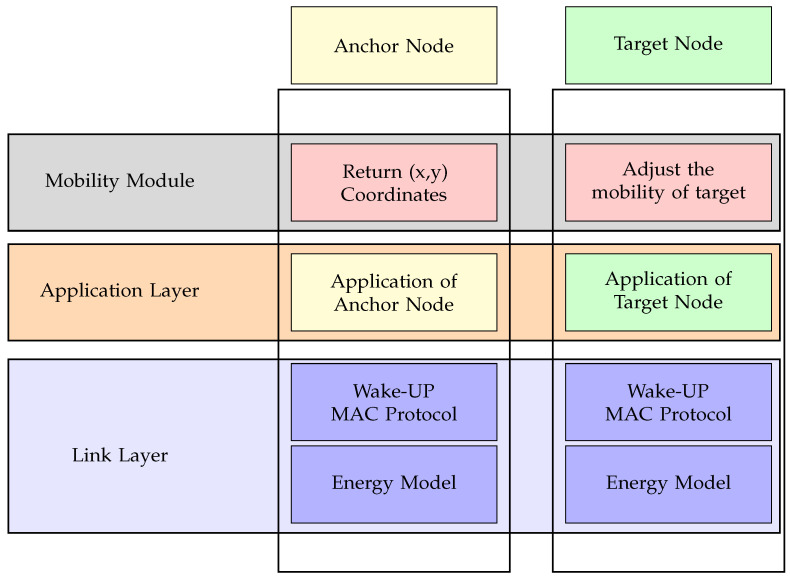
Architecture of used nodes.

The target node and the anchor node have the same protocol for the link layer, which is the wake-up MAC protocol as defined in the previous section. Both have the same energy model. The specific parameters of the energy model will be presented in Section 5.2. Their mobility is defined by the Mobility submodule. It handles the mobility for the target node and It keeps the anchor nodes in fixed coordinates. Figure 6 illustrates the similarities and distinctions in the architectures of anchor nodes and target node.

The developed positioning system begins with initialization, where a target device within the network attempts to find its position. The tested environment is covered by anchors. These anchors play a critical role in assisting the target device in its positioning task. The first step is for the target device to send out a wake-up call. This wake-up call is used to activate the anchor node and establish communication channels within the network. The target then waits for replies from anchors. The next phase focuses on receiving acknowledgment signals, commonly referred to as acknowledgement (ACK), from anchor nodes. The target device anticipates the receipt of three different ACKs, interpreting this response as a successful wake-up of three anchors. Once the required number of ACKs is received, the target device proceeds to transmit a signal. Then, it enters a waiting phase in which it awaits a signal from the three closer anchor nodes. This signal contains vital information, including the positions of the anchor nodes and the distances between them and the target. Typically, this information is extracted from RSSI measurements, which help to estimate spatial relationships within the network. If the target does not receive the expected signal with position and distance information, it returns to the initialization state and increases its transmission power by 5 dBm. Following the adjustment in transmission power, the target device retransmits the signal, ensuring that it reaches its intended destination.

**Figure 7:**
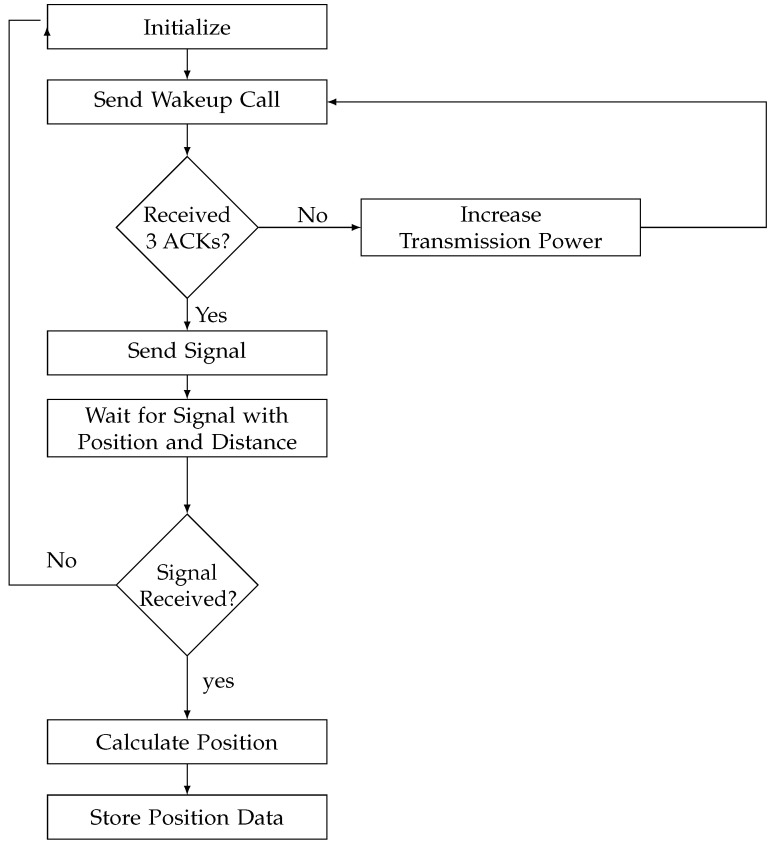
Flow chart of the proposed algorithm.

After successful reception from desired anchors, another signal with anchor position and distance extracted from RSSI of the received signal will be transmitted. The extraction of the distance information and the preparation of the signal information are done by the application layer of the anchor nodes. The information is transmitted to the target node by the link layer. The target device can proceed with the calculation of its position. This calculation is typically based on trilateration techniques using the known positions of the anchor nodes and the measured distances between them and the target. Finally, calculated position information is stored locally in the target memory. The flowchart of this algorithm is presented in Figure 7.

## 5. Simulation and Results

This research presents a precise algorithm for indoor localization applications, focusing on the challenges of extending the lifetime of wireless sensor networks and evaluating the accuracy of the presented algorithm. The main challenge is to deal with real-world parameters to simulate large-scale indoor localization scenarios. To address these complexities and improve the efficiency of the Wireless Sensor Network (WSN) design, an innovative approach is taken: simulating the network through OMNeT++. The main goal is to create a simulated environment that accurately reflects the behavior of the WSN under various conditions. Taking advantage of OMNeT++, a simulation is developed using the wake-up MAC protocol with a detailed application. A special C++ code is developed that allows exploration, evaluation, and optimization of the performance of the WSN before proceeding to real-world implementation.

Furthermore, it enables us to fine-tune critical parameters, such as transmission power, and node placements, to achieve optimal system performance and energy efficiency. This work also involves verification of scalability tests, allowing us to understand how WSN will perform as the network’s size or coverage area expands. Additionally, collects invaluable performance metrics such as accuracy and energy consumption, during simulations, thus facilitating informed decision-making and realistic expectations for the real-world deployment.

Similarly, different parameters are analyzed, including worst-case and edge-case scenarios, to assess how WSN responds to various conditions, such as interference and mobility patterns. Additionally, OMNeT++ enables us to develop and validate localization algorithms, and data aggregation strategies, ensuring their correctness and efficiency within the controlled simulation environment. In summary, the approach of employing OMNeT++ for simulating WSN underlines the commitment to thorough and systematic research and development.

### 5.1. OMNeT++

OMNeT++ discrete event simulator is a widely used framework for modeling and simulating complex communication networks and distributed systems. The use of OMNeT++ as a simulation tool forWSNs is a valuable strategy, especially when dealing with uncertain factors and aiming to evaluate system performance before real-world deployment. By developing C++ code within OMNeT++, researchers and engineers can create a virtual environment that reflects the behavior of their planned WSN in various scenarios.

These include reducing risk by identifying potential problems without the cost of physical implementation, optimizing parameters, facilitating scalability testing, enabling comprehensive performance assessment, supporting scenario-based testing to assess system robustness, supporting algorithm and protocol development, and enabling rigorous validation of custom communication protocols.

By utilizing OMNeT++ in this manner, project teams can make informed decisions, fine-tune system parameters, and enhance the likelihood of a successful real-world WSN implementation. It is an open source based on components for education and research purposes. It is a component-based, modular, and open-architecture simulation environment with strong graphical user interface (GUI) support and an amendable simulation kernel. It is an object-oriented discrete event simulation framework. Its primary application area is the simulation of communication networks on a large scale, but it has been successfully used in other areas like the simulation of IT systems, queuing networks, hardware architectures, and business processes as well [34].

### 5.2. Energy Parameters

**Figure 8:**
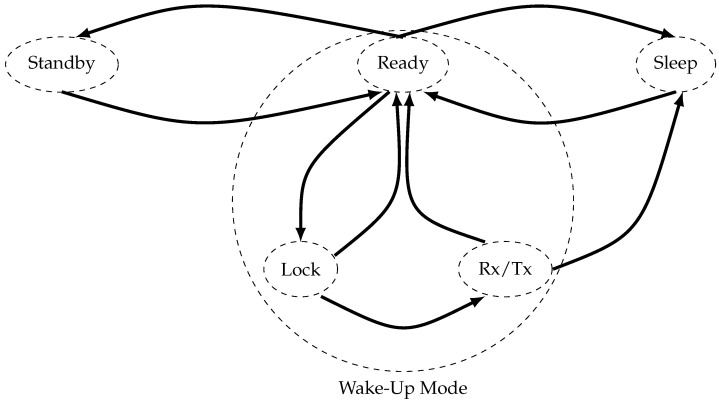
Machine states radio mode.

The proposed model uses the energy states of the hardware AS3933 and Spirit1 low data and low power data according to [35]. According to Figure 8, it contains five radio modes in relation. Each mode presents a different energy consumption [35]. To change from ready to receiving or transmitting mode it should pass through lock mode. For our model, we assume the change of modes from ready to Receiving or transmitting mode to wake-up mode. Table 5 presents the energy consumption of each mode. The main goal of studying the different states of the radio transceiver is to approach the real power consumption in the simulation.

### 5.3. Estimation of Exponent Path-Loss Model

**Figure 9:**
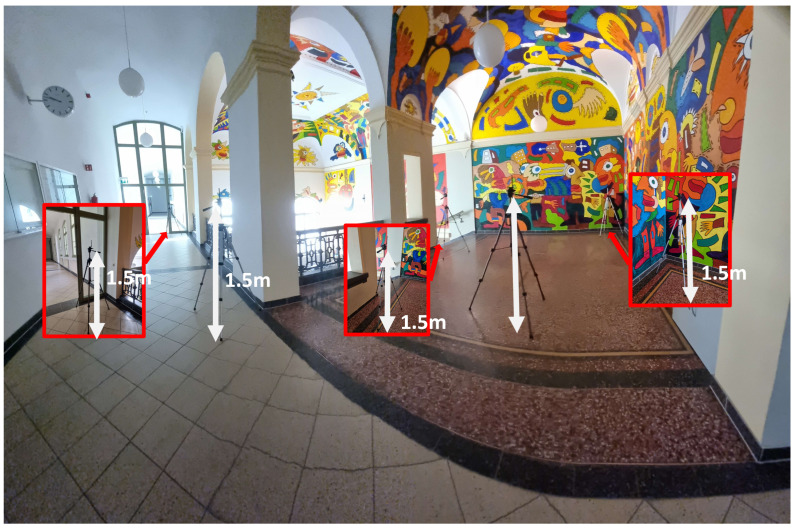
The actual measurement conditions within the corridor of HTWK University.

To use real parameters, we have attempted to estimate the RSSI in our environment, which is the most critical parameter in our simulation, to achieve accuracy close to reality. To use real parameters, we estimate the path loss exponent η and σ that describe our environment. To characterize the RSSI model in an indoor environment, an experimental setup was performed. Figure 9 presents the experimental environment. A WuRx node operating at 868 MHz frequency is used.

**Figure 10:**
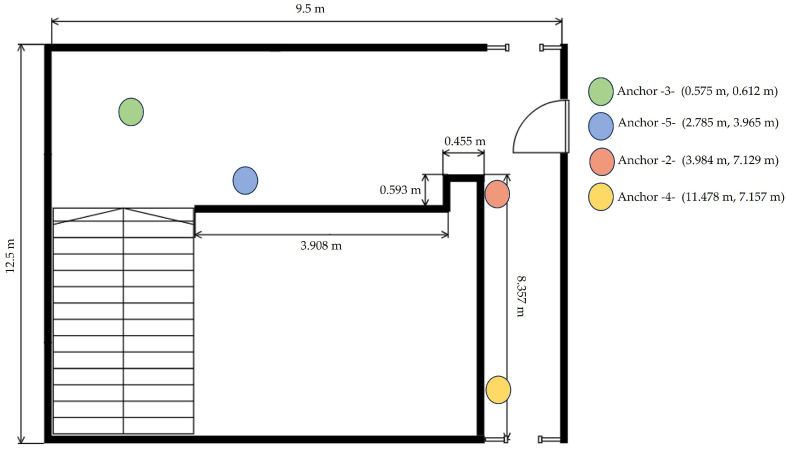
Measurement environment.

The main goal was to determine the parameters of the propagation model that best describe our real environment. Two of these parameters are the path loss exponent η and σ, which characterize the signal attenuation as a function of distance between nodes in a specific environment. In this context, four nodes have been used to collect RSSI data. These nodes have been strategically positioned within a corridor, maintaining a height of 1.5 m above the ground, to create a two-dimensional (x,y) plane. Our objective revolves around studying the behavior of a mobile target within this environment, with the target’s transmission power set at −15 dBm. The anchors were placed in different positions as shown in Figure 10.

Several samples of RSSI are collected using the putty tool. According to the different movements of the target inside the corridor, different data sets present RSSI values for each anchor and the distance calculated between the target and anchor. For such position of the target, we take measurements of 1 min. For estimation, a Python code is used with fitting techniques to find the values of path loss exponent η and σ values.

**Figure 11:**
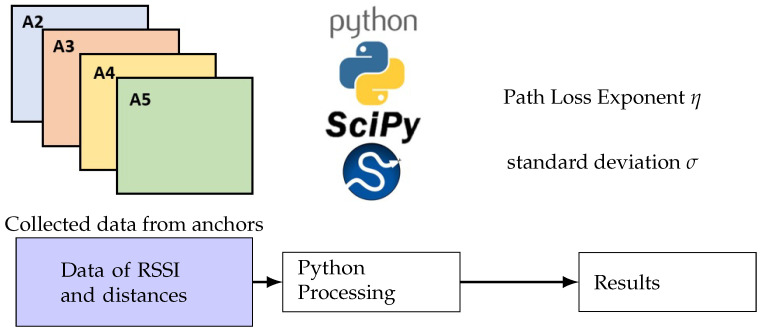
Data processing flow.

**Figure 12:**
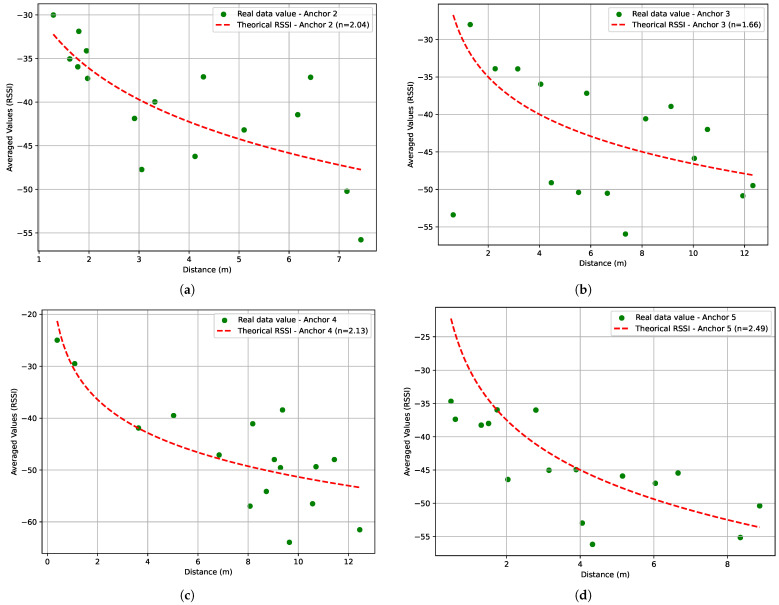
Real data RSSI values and theoretical RSSIs vs. distances for the selected anchors. (**a**) Real RSSI values and estimated path-loss model for different distances for anchor 2. (**b**) Real RSSI values and estimated path-loss model for different distances for anchor 3. (**c**) Real RSSI values and estimated path-loss model for different distances for anchor 4. (**d**) Real RSSI values and estimated path-loss model for different distances for anchor 5.

Figure 11 illustrates the coding process in which we used the SciPy library to estimate the parameters. $RSSI_{0}$ and $d_{0}$ are defined as −30 dBm and 1 m. Table 6 present above showcases the parameters of various anchor nodes, including η and $X_{\sigma }$. These values provide valuable information on the characteristics of the anchor nodes within the network, helping to understand the propagation of the signal and the environmental factors that influence wireless communication. Figure 12a–d shows the result of theoretical RSSI using the path loss exponent η mentioned in Table 6 and the real RSSI data measurements collected from anchors 2, 3, 4 and 5.

**Figure 13:**
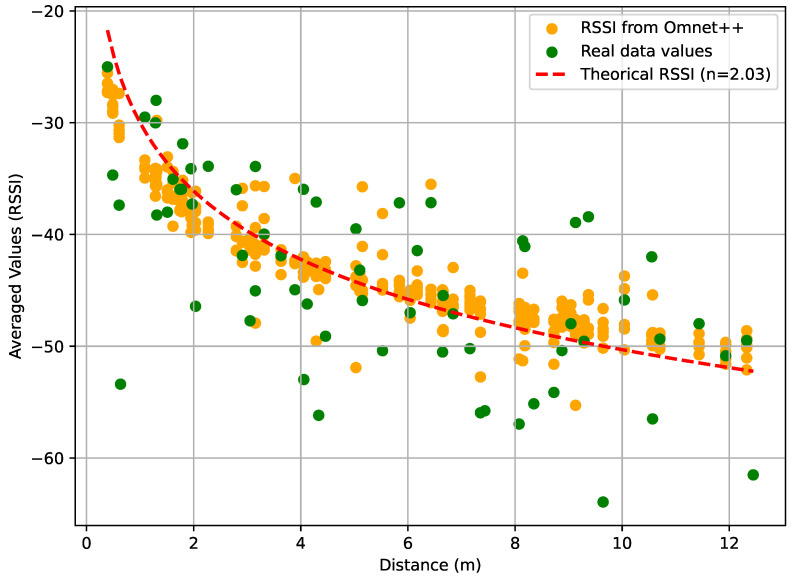
Received signal strength indicator measurements real data vs. estimated data and data generated by OMNeT++.

Next, we use the data collected from all anchors to estimate the values of the parameters η and σ, which indicate the prevailing environmental conditions. We find *n* equal to 2.03 and σ equal to 1.83 dB with a mean RSSI equal to −43.75 dBm. Meanwhile, we performed the implementation of the actual target path within the OMNeT++. This implementation was carried out in conjunction with the anchor positions and allowed us to generate RSSI data based on the corresponding distances between the target and the anchors. Figure 13 shows the results of the estimated RSSI for the entire data set and the data generated by OMNeT++ under the same environmental conditions. The RSSI implemented used a Cauchy distribution with a mean −43.75 dBm and a standard deviation equal to 1.83 dB. Equation (15) presents the probability density function of the Cauchy distribution [36]:

(15) $$f\left(x\right)=\frac{1}{\Pi }\frac{\gamma }{{\left(x-a\right)}^{2}+\gamma^{2}}$$

where −∞<x<∞ and cumulative distribution function is

(16) $$F\left(x\right)=\frac{1}{2}+\frac{1}{\Pi }\arctan\left(\frac{x-a}{\gamma }\right)$$

The output values are generated by the Cauchy distribution from the continuous distribution of the OMNeT++ simulation library. The library provides a function that returns a Cauchy random variate using the values of mean RSSI −43.75 dBm and the standard deviation 1.83 dB. To evaluate the effectiveness of our application, we used the true path and the distribution derived from OMNeT++ to estimate the different positions of the target. The resulting root mean square error (RMSE) was 1.79 m for the localization of 15 different positions. Based on these results, the proposed application demonstrates a lower error rate within a dimension cell 12.5 m × 6 m compared to state-of-the-art methods presented in [17,18]. These methods, which use trilateration or multilateration, have higher errors using cells of 5 m × 5 m and 9.77 m × 13.45 m, respectively. They present 3.6 m and 2.33 m as the average error for a smaller and similar cell when the proposed method presents 1.58 m. Based on the confirmed results for a single cell, we extend the studied area to 40 m × 40 m with a 20 m × 20 m cell size in the next section.

### 5.4. Localization Accuracy

In this section, we will use the RSSI estimation function, the energy model, the wake-up MAC protocol, and the matrix architecture defined in the previous sections to facilitate the simulation of a real-world mobile target localization environment within the OMNeT++. This simulation phase precedes the eventual hardware implementation. To achieve this, we will rely on the RSSI data estimated in the previous section. Table 7 details the simulation parameters.

**Figure 14:**
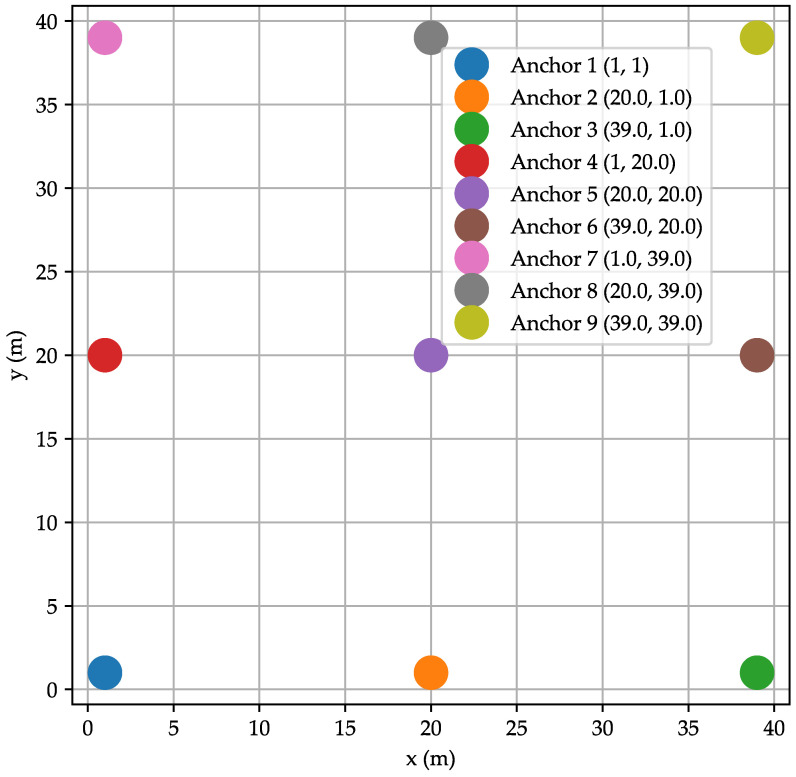
Area architecture.

The architecture of the network and the arranging of anchors are detailed in Figure 14. The area will be divided into four cells. Cells 1 and 2 are covered by anchors 1, 2, 4, 3, 5, and 6, while cells 3 and 4 are covered by anchors 4, 5, 6, 7, 8, and 9. Table 7 presents the parameters of the simulation. The area is 40 m × 40 m, and each cell with 20 m × 20 m. The target and the anchors are equipped with two AA batteries with a capacity of 2790 mAh. The application will be used for the localization of 100 positions. The initial value of the transmission power of the target node starts from −15 dBm and is incremented by 5 dBm if three acknowledgments do not appear, as mentioned in the application flow chart of Figure 7. To perform trilateration calculations, the target was equipped with an MSP430 MCU from Texas Instruments technology. These types of MCU are known for their ultralow power consumption. The mobility of the target is along an arbitrary and rather irregular path in space with a variable speed. The nominal speed of the target is 1.3 m·s^−1^.

As discussed in the previous paragraph, RSSI measurements are sensitive to environmental conditions and noise. The RSSI serves as a critical parameter for our trilateration method. To evaluate the accuracy of our model, we introduce variations in the exponent path loss model η to evaluate the accuracy of our model.

To analyze the results obtained, we used the parameters root mean square error (RMSE) and mean Squared Error (MSE). The RMSE has been used as a standard statistical metric to measure the performance of the model in meteorology, air quality, and climate research studies. The mean absolute error (MAE) is another useful factor widely used in model evaluations [37].

(17) $$E_{i}={\left(y_{i}-{\hat{y}}_{i}\right)}^{2}$$

where:

- $E_{i}$ is the squared error for data point *i*.
- $y_{i}$ is the observed (actual) value for the data point (i).
- ${\hat{y}}_{i}$ is the predicted value for data point *i*.

(18) $$MSE=\frac{1}{n}\sum_{i=1}^{n}E_{i}$$

where:

- RMSE is the mean (average) of the MSE squared errors.
- *n* is the total number of data points.

(19) $$RMSE=\sqrt{\mathrm{MSE}}$$

**Figure 15:**
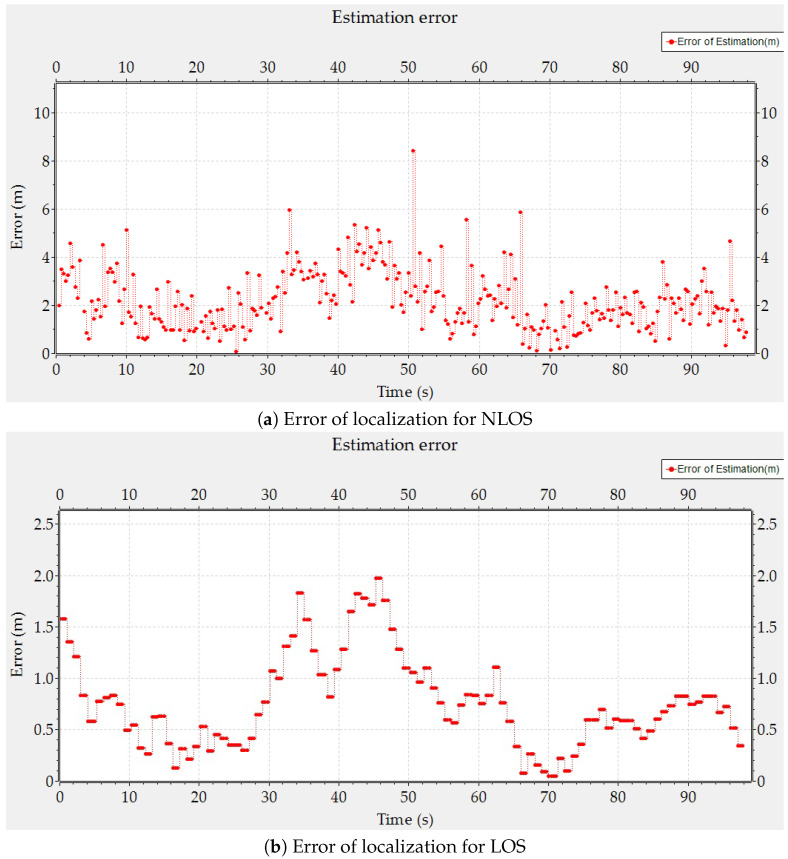
Error of localization for NLOS and LOS.

As shown in Figure 15, the proposed application shows a high performance and reliability of the application with the two environments in different conditions of line of sight (LOS) and non line of sight (NLOS). Figure 15a presents an RMSE equal to 2.52 m for NLOS conditions. Signal measurements include an error due to the additional path traveled. Almost 90% of errors are between 0.07 m and 3.79 m. The average error for 90% is 1.91 m. The proposed application reflects peaks at 8 m and 6 m resulting from RSSI deviation in response to real-world environmental factors; this is often referred to as non-line-of-sight (NLOS) conditions. For the LOS, the proposed application presents the best performance by an RMSE equal to 0.88 m. Figure 15b presents the error of localization according to LOS conditions. 90% of errors are between 0.05 m and 1.41 m, for an average of 0.65 m.

**Figure 16:**
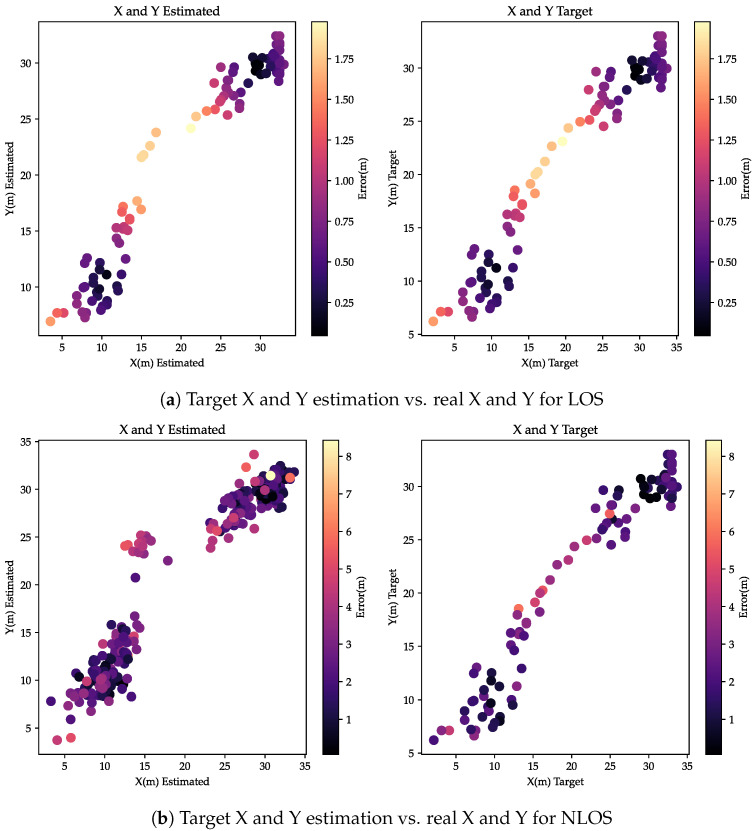
Target X and Y estimation vs. real X and Y for NLOS and LOS.

Figure 16 compares the estimated target path and the actual path for both environment conditions LOS and NLOS. Figure 16a shows the estimated path with the real path according to the error of localization. The curve of the estimated path shows the same shape with a deviation of 4 m in the middle of the area for an NLOS and 1.75 m for an LOS. According to Figure 16a,b the proposed application can localize the target during its mobility path without position loss.

**Figure 17:**
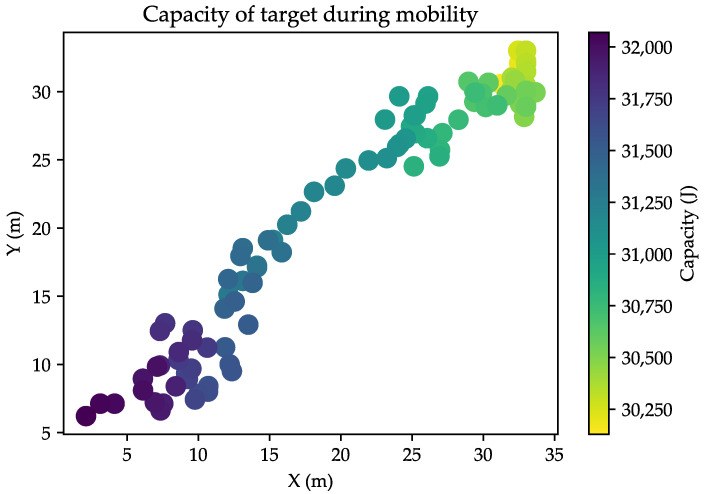
Energy consumption of target according to communication under mobility.

To evaluate the effectiveness of the proposed method, we performed simulations to analyze the energy consumption of both the anchors and the target within our application. The energy capacity of all nodes is quantified in joules J. The initial capacity is 32,076 J for all used anchors and the target. This capacity is obtained according to the conversion from mAh to J. Figure 17 shows the energy consumption of the target relative to its mobility path and self-localization application. The energy is decreased according to the mobility of the target due to the communication. After locating 100 positions using our application, the capacity of the target decreased to 93.95%. Based on the consumption information provided, if the power is consumed continuously for the localization of 100 positions monthly, the battery would be depleted after the localization of 1653 positions.

**Figure 18:**
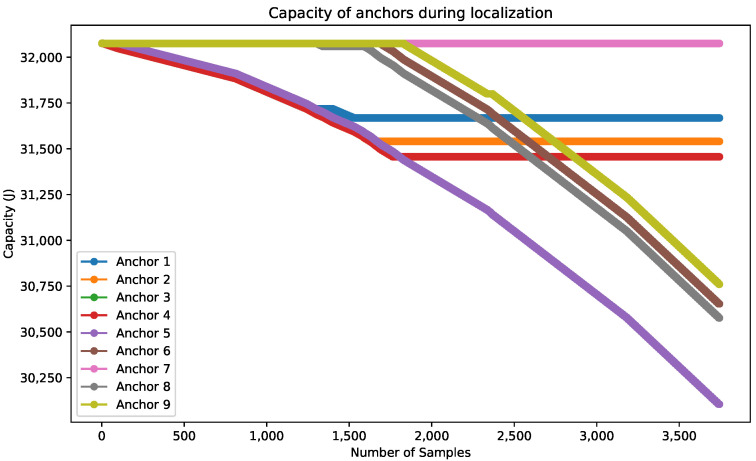
Capacity of anchors according to localization application.

For the remaining anchors, their energy consumption varies depending on their communication with the target within the localization application. The proposed application is based on dynamic anchors to reduce energy consumption during localization. Table 8 shows the percentage of battery consumption after localization of 100 positions with the consumption in J. The total network consumption is about 2.69%. Anchors 3 and 7 remain unused. Figure 18 shows the consumption during the localization application. At the beginning, the target moves under the cell covered by anchors 1, 2, 4, and 5. From this cell, just anchors 1, 2, and 4 are used for the localization during the first positions. When the target moves closer to anchor 5, anchor 1 switches to sleep mode, and anchors 5, 2, and 4 perform the localization. When our target moves to cell number 4 covered by anchors 5, 6, 8, and 9, anchors 2 and 4 switch to sleep mode, while anchors 5, 6, 8, and 9 are awakened according to the position of the target. Figure 18 shows how anchors 1, 2, and 4 consume energy until they switch to sleep mode, and anchors 6, 8, and 9 are awakened by the target for the latest position.

### 5.5. Variation in Exponent Path-Loss Model

The simulation was kept under the same conditions, with variations limited to the value of η, specifically at 3.0. It was observed that the increase in the attenuation factor η leads to an increase in error, as evidenced by the data collected. Nevertheless, our application continued to show efficiency at different levels.

Setting η to 1.6 or less consistently produces the best results. Increasing η directly affects the RSSI and subsequently increases the complexity of the trilateration calculations, primarily due to interference from obstacles. However, It is important to note that changing the η value has a significant impact on the error estimates and it is due to the difference between the type of environment. Table 9 shows the difference between RMSE for LOS and NLOS. Table 10 presents an error classification according to framing between 1 m and 2 m. The majority of errors are up to 2 m for η equal to 3. For η equal to 2.03 presents different levels of error classification by 34.02% between 1 m and 2 m, 49.14% for errors up to 2 m and 16.84% for error less than 1 m. These illustrations effectively demonstrate how the presence of obstructions and the resulting NLOS conditions significantly affect the reliability and accuracy of RSSI-based location techniques. However, the proposed application according to the dynamic selection reduces the effect of obstacles compared to other studies.

**Figure 19:**
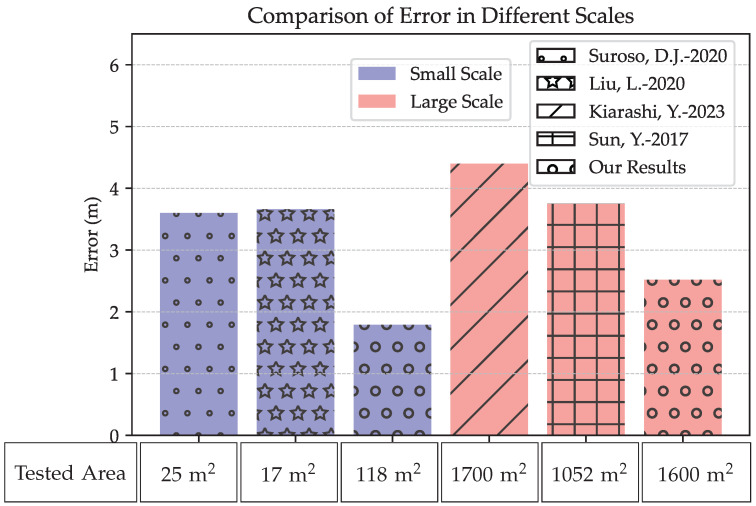
Comparisonof our work results with the state of the art in different scale areas; Suroso & Arifin, 2020 [17]; Liu et al., 2020 [38]; Kiarashi et al., 2023 [39]; Sun et al., 2017 [40].

Compared to other results for a large-scale area, the proposed method presents an effective RMSE for non-line-of-sight conditions. A comparison of our results in terms of error and tested area with the various states of the art is shown in Figure 19. For a small-scale area, the authors of [17] used ZigBee technology with RSSI for an area of 25 m^2^ and they present an average error of 3.6 m. In [38], the authors used the iBeacon method with a trilateration algorithm and a specific fingerprinting method. They present an error of 3.66 m for an area of 17 m^2^. The proposed methods present performed results for a small area scale by 1.87 m and 1.81 m compared to results presented in [17,38], respectively. In [39], the authors used an area of 1700 m^2^ in a complex environment and presented an average error of 4.4 m with BLE technology, which costs more than the technology used and has lower range detection. For [40], the authors proposed optimized propagation model parameters in order to optimize parameters for trilateration localization. Their experiments covered a space of 1052 m^2^ and yielded an average error of 3.75 m. Comparatively, the proposed approach demonstrated superior performance in larger areas, showing improvements of 1.23 m and 1.88 m, respectively, for similar spatial ranges [39,40]. Balancing the need to localize in all positions with energy efficiency remains challenging. The proposed application presents significant results for energy consumption and accuracy for different environments.

## 6. Conclusions

Energy saving and accuracy are critical issues for indoor localization applications due to high communication between nodes and interference with other signals. This challenge is particularly pronounced in large-scale WSN. To address these issues, we present a novel indoor localization application that uses RSSI and trilateration for WSN. The goal is to achieve optimal localization results while extending the lifetime of sensor networks.

The application of localization is related to many factors to decrease the energy consumption of the network. A new application is developed with a variation in power transmission to reduce the number of anchors used. To assess the reliability of our application, we have incorporated real-world parameters into our network simulations. The fixed RSSI model is extracted from real data, ensuring a true reflection of the behavior and performance of the network. The goal of this study is to evaluate the lifetime and precision of indoor localization applications to identify optimal performance conditions referring to real parameters. Our solution provides an impressive accuracy of approximately 2.52 m for a consumption of 2.69% for a large area scale and 1.79 m for a small area scale. Under different environmental conditions, the proposed application in this research demonstrates impressive accuracy. It provides an accuracy of 0.88 m under line-of-sight (LOS) conditions and 3.05 m in the most challenging scenarios of non-line-of-sight (NLOS) conditions. Simulation results show that the proposed algorithm in this study achieves superior accuracy compared to various studies. Based on the obtained results under different conditions and parameter variations between line-of-sight (LOS) and non-line-of-sight (NLOS) scenarios, covering both large and small areas, as well as the validation in terms of accuracy and energy consumption of both anchors and target nodes, our future endeavors include the implementation of the application in a real network for a comparative analysis with the presented results.

## Figures and Tables

**Table 1:** Summary of technology approaches and corresponding accuracies according to tested area and localization technique.

Reference	Technology	Localization Technique	Tested Area	Average Error
[17]	ZigBee	RSSI	5 m × 5 m	3.6 m
[18]	BLE	RSSI Multilateration+ Kalman Filter	9.77 m × 13.45 m	2.33 m
[19]	LoRaWAN	RSSI Trilateration	5.6 m × 5.9 m	1.025 m
[20]	UWB	TOF	12 m × 8 m	1.56 m
[21]	RFID	RSSI	2.46 m × 4 m	0.18 m
[22]	Infrared	Neural networks	3 m × 3 m	0.096 m
[23]	Ultrasound	TOF	1.2 m × 1.2 m	0.012 m

**Table 2:** Wake-up receiver hardware description.

Hardware Component	Detailed Description
Wireless transceiver	Spirit1 SPS-GRFC-868 Sub-GHz transceiver
MCU	MSP430G2553 Serie 16-Bit microcontroller
Sensors	LSM9DS1, Si7021
RF Bandpass Filter	B39871B3725U410
Envelope Detector	Greinacher Voltage Double, SMS7630-006LF
LF Amplifier	Single stage BJT amplifier, BFP 405
LF pattern matcher (WuRx)	AS3933

**Table 3:** Path Loss Exponent η for different indoor environments.

Environment Type	Range Value of Path Loss Exponent η
Buildings, Case Line of Sight	1.6 to 1.8
Factory obstructions	2 to 3
Building obstructions	4 to 6

**Table 4:** Signal Strength levels.

RSSI (dBm)	Signal Strength
>−50	Excellent
−50 to −60	Good
−60 to −70	Fair
<−70	week

**Table 5:** Energy consumption according to the different states of the radio module.

Simulation Parameters	Values
StandBy	1.98 μW
Ready	1.32 mW
Lock	14.52 mW
Rx	32.01 mW
Tx	69.3 mW
Sleep	2.805 μW

**Table 6:** Values of η and σ for each anchor.

Anchor Node	η	σ (dB)	Mean RSSI (dBm)
Anchor 2	2.04	1.78	−40.13
Anchor 3	1.66	2.04	−43.56
Anchor 4	2.13	1.92	−46.94
Anchor 5	2.49	1.81	−44.42

**Table 7:** Simulation parameters.

Parameters	Values
Area	40 m × 40 m
Anchor number	9
Battery	2× AA battery 2970 mAh at 1.5 V
Number of positions	100

**Table 8:** Energy consumption of each node.

Anchors	Percent of Consumption	Consumption in (J)
Anchor 1	1.27%	408
Anchor 2	1.67%	536
Anchor 4	1.93%	620
Anchor 5	6.14%	1971
Anchor 6	4.43%	1422.04
Anchor 8	4.68%	1500
Anchor 9	4.10%	1316

**Table 9:** Comparison between MSE and RMSE in LOS and NLOS with the variation in η.

η Values	MSE	RMSE	Average Error	Type of Environment
1.6	0.77	0.88	0.76	LOS
2.03	6.35	2.52	2.19	NLOS
3.0	9.32	3.05	2.59	NLOS

**Table 10:** Error classification.

η Values	Error < 1 m	Error between 1 and 2 m	Error > 2 m
1.6	74.41%	25.59%	0%
2.03	16.84%	34.02%	49.14%
3.0	12.46%	29.63%	57.91%

## Data Availability

Data are contained within the article.

## Abbreviations

The following abbreviations are used in this manuscript:

FM	frequency modulation
RFID	radio frequency identification
IoT	internet of things
OMNeT++	objective modular network testbed in C++
WSN	wireless sensor network
MAC	media access control
GPS	global positioning systems
RSSI	received signal strength indication
AOA	angle of arrival
WuRx	wake-up receiver
RMSE	root mean square error
MAE	mean absolute error
MSE	mean Squared Error
RF	radio frequency
TOF	time of flight
TDOA	Time Difference of Arrival
UWB	ultra wide band
MCU	microcontroller unit
WuPt	wake-up packet
ACK	acknowledgement
GUI	graphical user interface
LOS	line of sight
NLOS	non line of sight
